# Highly Efficient Catalytic Performances of Nitro Compounds and Morin via Self-Assembled MXene-Pd Nanocomposites Synthesized through Self-Reduction Strategy

**DOI:** 10.3390/nano9071009

**Published:** 2019-07-12

**Authors:** Juanjuan Yin, Lun Zhang, Tifeng Jiao, Guodong Zou, Zhenhua Bai, Yan Chen, Qingrui Zhang, Meirong Xia, Qiuming Peng

**Affiliations:** 1 State Key Laboratory of Metastable Materials Science and Technology, Yanshan University, 438 West Hebei Street, Qinhuangdao 066004, China; 2 Hebei Key Laboratory of Applied Chemistry, School of Environmental and Chemical Engineering, Yanshan University, 438 West Hebei Street, Qinhuangdao 066004, China; 3 National Engineering Research Center for Equipment and Technology of Cold Strip Rolling, Yanshan University, 438 West Hebei Street, Qinhuangdao 066004, China

**Keywords:** nanocomposites, catalyst, MXene, self-reduction, palladium nanoparticles

## Abstract

With development of the society, the problem of environmental pollution is becoming more and more serious. There is the urgent need to develop a new type of sustainable green material for degradable pollutants. However, the conventional preparation method is limited by conditions such as cumbersome operation, high energy consumption, and high pollution. Here, a simple method named self-reduction has been proposed, to synthesize highly efficient catalytic nitro compounds and morin self-assembled MXene-Pd nanocomposites. Palladium nanoparticles were grown in situ on MXene nanosheets to form MXene@PdNPs. MXene@PdNPs composites with different reaction times were prepared by adjusting the reduction reaction time. In particular, MXene@PdNPs20 exhibited a high catalytic effect on 4-NP and 2-NA, and the first-order rate constants of the catalysis were 0.180 s^−1^ and 0.089 s^−1^, respectively. It should be noted that after eight consecutive catalytic cycles, the conversion to catalyze 4-NP was still greater than 94%, and the conversion to catalyze 2-NA was still greater than 91.8%. Therefore, the research of self-assembled MXene@PdNPs nanocomposites has important potential value for environmental management and sustainable development of human health, and provides new clues for the future research of MXene-based new catalyst materials.

## 1. Introduction

With development of the society, the problem of environmental pollution has become more and more serious, and the type and number of pollutants have been increasing continuously, especially chemical dyes that are indispensable in the chemical industry. However, these pollutants pose a great threat to the social environment and human health. Therefore, there is an urgent need to develop a new type of biodegradable green materials at present.

In recent years, researchers have made many efforts in the field of dye degradation. For example, Zhang et al. prepared a composite material, the CaMoO_4_/conductive polymer, which is relatively ecologically efficient and can be used for the recovery of silicomanganese slag and the degradation of dye wastewater [1]. Kampouri et al. used MIL-125-NH_2_ under visible light irradiation for photocatalytic hydrogen production and dye degradation [2]. In addition, since 2011, Gogotsi and coworkers reported a two-dimensional (2D) transition metal carbide or carbonatite (MXenes) [3]. Because MXenes materials have excellent surface chemistry, high conductivity, excellent ductility and flexibility, they are widely studied in the fields of energy storage and conversion, environmental adsorption, and wastewater treatment [4,5,6,7,8,9].

At present, our group has prepared new composite materials such as MXene/magnetic iron oxide nanocomposite, which have good electrochemical catalytic activity, excellent dye catalytic performance and sodium aluminate dehydrogenation catalytic activity [10,11,12,13]. The preparation of MXene-based composites using simple, highly efficient self-assembly methods remains a significant challenge. The Pd catalyst used in this experiment not only catalyzes nitroaromatic compounds, but also has better activity, robust stability and magnetic recyclability. For examples, Huang et al. successfully prepared novel AgNPs-loaded PVA/PAA/Fe_3_O_4_/MXene composite nanofiber materials by electrospinning and self-reduction of AgNO_3_ for catalytic degradation of nitroaromatic compounds [14]. Wang et al. prepared polyethyleneimine/polycaprolactone/palladium nanoparticle composite fiber membranes for catalyzing a nitro compound with high catalytic performance and recyclability [15]. Zhang et al. prepared well-dispersed Ni nanoparticle in situ on silica nanotubes with excellent catalytic activity in 4-nitrophenol reduction [16]. The in-situ growths of gold nanoparticles on MnO_2_ nanosheets were studied to form Au@MnO_2_ nanocomposites for reduction of 4-nitrophenol (4-NP) and methylene blue (MB) [17]. In this paper, novel MXene@PdNPs nanoparticle composites were synthesized on the layered MXene (Ti_3_C_2_(OH_x_F_1−x_)_2_) used in the template, and its morphology was characterized. Catalytic degradation of compounds such as nitroaniline (2-NA), 4-nitrophenol (4-NP) and morin were further investigated. In addition, we also investigated the catalysis of nanocomposites prepared at different reaction times for morin. Study results have shown that MXene@PdNPs composites have good catalytic efficiency, catalyst stability and high recyclability. Present research work provides broad application prospect for MXene-based composite materials in the field of new catalysts for dye catalytic degradation and sewage treatment.

## 2. Materials and Methods

### 2.1. Materials

2-NA and 4-NP were purchased by Shanghai Aladdin reagent factory. The ultra-pure water used in the course of the experiment was prepared using the Milli-q microporous filtration system (Millipore, Bedford, MA, USA). The ethanol (C_2_H_5_OH) used in the experiment was supplied by Sinopharm Chemical Reagent Co., Ltd. (Beijing, China). MXene was synthesized according to literature report [18]. In addition, all the chemicals used in the experiment were directly used without further purification.

### 2.2. Preparation of MXene@PdNPs Composite

Briefly, MXene was prepared by adding an appropriate amount of 49 wt% HF to the Ti_3_AlC_2_ powder for 24 h at room temperature in order to etch away the Al layer in Ti_3_AlC_2_. After HF treatment, it was soaked in deionized water until the pH of the solution was greater than 6. Then, the sample was vacuum dried at 80 °C for 12 h to finally obtain MXene. First, 100 mg of MXene solid powder was added to 100 mL of ultrapure water and subjected to constant magnetic stirring. Next, the suspension was sonicated for one hour to obtain a uniform suspension. Thereafter, 3 mL of PdCl_2_ (0.1 mol/L) was gently added and stirring was continued to finally obtain a composite MXene@PdNPs. According to the time of reaction, it is divided into MXene@PdNPs5 and MXene@PdNPs20. The obtained complex was washed by centrifugation with ultrapure water several times and then freeze-dried for 48 h.

### 2.3. Catalytic Performance Test

The MXene@PdNPs suspensions (0.3 mL, 1 mg/mL) were mixed with 2-NA aqueous solution (2 mL, 5 mM) or 4-NP (2 mL, 5 mM), and fresh NaBH_4_ solution (20 mL, 0.01 M) was added. Finally, the sample solution was determined by UV-vis spectra until the mixed solution became colorless. In addition, catalytic mulberry pigments were also tested. First, a buffer solution of sodium carbonate and sodium bicarbonate (the ratio of the amount of the substance is 1:9) and a 3 mM morin solution (using a buffer solution as a solvent) was prepared. Next, 1800 μL of the buffer solution, 40 μL of mulberry solution and 50 μL of catalyst MXene@PdNPs (0.031 mg/mL) were sequentially added to the cuvette, and finally 80 μL (0.2 mol/L) of the newly prepared hydrogen peroxide was added. Analysis was done by ultraviolet-visible spectroscopy until the solution became colorless.

### 2.4. Characterization

The morphology of the samples prepared in the experiments was characterized by scanning electron microscopy (SEM, Hitachi S4800, and Japan) and transmission electron microscopy (TEM, HT7700, Hitachi high technologies Corporation, Ibaraki, Japan). Composite images were taken at 200 kV using JEM-2010 electron microscopy. High resolution transmission electron microscopy (HRTEM) images were acquired with a JEM-2010 electron microscope operated at 200 kV. X-ray diffraction (XRD) patterns of the samples were obtained with an X-ray diffractometer equipped with a Bragg diffraction setup (SMART LAB, Rigaku, Akishima, Japan) and a Cu Kα X-ray radiation source. At different times, the reaction solution concentration was measured by ultraviolet absorption spectroscopy using a UV-2550 spectrophotometer. X-ray photoelectron spectroscopy (XPS) analysis was performed on a Thermo ScientificESCALab 250Xi equipped with 200 W of monochromatic Al Kα radiation. In this experiment, the catalytic performance of the catalyst was investigated by UV-Vis spectroscopy (UV-2550, Shimadzu Corporation, Kyoto, Japan).

## 3. Results and Discussion

### 3.1. Structural Characterization of Nanocomposites

**Figure 1:**
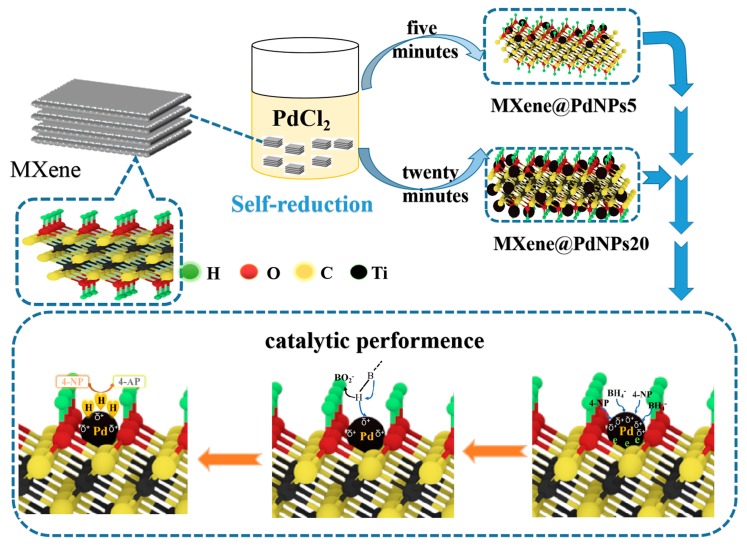
Schematic illustration of preparation and catalytic application of PMXene@PdNPs materials.

In this study, self-assembled MXene-Pd nanocomposites synthesized by a self-reduction strategy have high catalytic performance for nitro compounds and morin. First, a solution of PdCl_2_ (3 mL, 0.1 M) was slowly added to a suspension of MXene (100 mL, 1 mg/mL), and Pd^2+^ ions were reduced to form PdNPs, as shown in Figure 1. According to the reaction time, two kinds of MXene@PdNPs composites were produced, namely MXene@PdNPs5 and MXene@PdNPs20. Next, the catalytic activity of MXene@PdNPs for 2-NA, 4-NP and morin was studied. In this experiment, since the electrons in the low-valent titanium in MXene are oxidized to a high valence state, the electrons of the Pd^2+^ ions are reduced to PdNPs. On the other hand, MXene also provides an attachment site for Pd^2+^ reduction, so that the palladium nanoparticles can be well distributed on the surface of MXene, thereby reducing the aggregation of palladium. The catalytic mechanism for the catalytic reduction of p-nitrophenol is the transfer of electrons from the electron donor BH_4_^−^ to the electron acceptor 4-NP. Specifically, in the solution, BH_4_^−^ ions are first adsorbed on the surface of the catalyst. In the presence of BH_4_^−^, the 4-NP molecule decomposes and forms 4-nitrophenol ions due to the weak basicity of the sodium borohydride solution. After electron transfer to the metal catalyst, electrons of some PdNPs migrate to the MXene carrier, and the electron density of the PdNPs is changed by electron transfer. In addition, after electron transfer to the metal catalyst, hydrogen ions in the hydride spontaneously attacks the 4-NP molecule, and finally results in the in situ hydrogenation reaction of 4-NP with active H produces 4-AP [19]. Similarly, 2-NA contains a nitro group, which requires catalytic hydrogenation to be converted to an amino group like catalytic process of 4-NP, thus indicating that the catalytic mechanism of 4-NP and 2-NA is consistent. Since MXene-Pd nanocomposites have catalytic properties for 4-NP, they also have corresponding catalytic properties for 2-NA. The MXene-Pd nanocomposite has a good catalytic effect on the nitro compound because it catalyzes the hydrogenation of the nitro group into the amino group [20].

**Figure 2:**
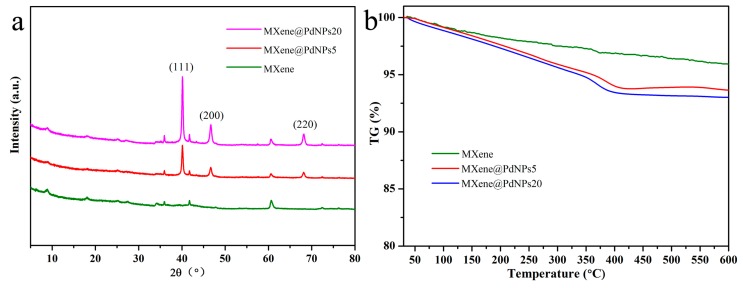
XRD patterns (**a**) and TG curves (**b**) of prepared MXene composites.

The prepared MXene, MXene@PdNPs5 and MXene@PdNPs20 nanocomposites were further characterized by X-ray diffraction spectroscopy, as shown in Figure 2a. As can be seen from the three curves, there are mainly three diffraction peaks on each curve, namely (111), (200) and (220), which are attributed to palladium nanoparticles [21]. It is indicated that the palladium nanoparticles are successfully attached to MXene [22]. In addition, as the reaction time increases, it can be seen from the figure that the diffraction peak of palladium becomes sharper and the intensity of the characteristic peak increases, further indicating that the crystallization of palladium is getting better and better. But the number decrement of palladium nanoparticles leads to changes in the crystal structure of MXene [23].

Thermogravimetric analysis (TG) is used to analyze the thermal stability of nanocomposites. It is an important technology for studying functional nanocomposites [24,25,26,27,28]. In order to study the thermal stability of the prepared MXene and MXene@PdNPs nanocomposites, the thermal properties of the obtained nanocomposites were characterized by thermogravimetric analysis. MXene and MXene@PdNPs nanocomposites were tested under nitrogen conditions, as shown in Figure 2b. The obtained three TG curves show that as the temperature increases, the heat loss of the material increases gradually [29]. When the temperature is 400 °C, the heat loss of the material is the largest—about 7.5%. After that temperature point, the loss tends to be flat. As shown in Figure S1b, the TG curve of MXene@PdNPs60 shows similar change tendency, indicating similar thermal stability of the synthesized composite materials. 

**Figure 3:**
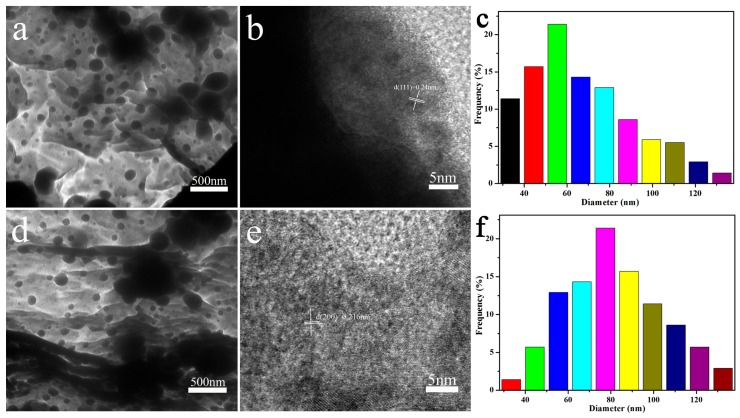
TEM images of the prepared MXene@PdNPs5 and MXene@PdNPs20 samples at 5, 20 min (**a,d**) and high-resolution TEM images of Pd nanoparticles (**b**,**e**), respectively and (**c**,**f**) Particle size distribution of Pd nanoparticles.

To further observe the structure of the prepared MXene@PdNPs complex, the TEM morphology is shown in Figure 3. The HRTEM image shows that the nanoparticles are single crystals. The interlunar spacing of (111) of pure PdNPs is 0.2243 nm (Figure S2). During the reduction of Pd by MXene, Pd particles grow slowly as the reaction time increases. The plane spacing d (111) = 0.240 nm and d (200) = 0.216 nm of the lattice data are clearly visible [20], as shown in Figure 3b,e. MXene is a new two-dimensional layered structure with higher visibility [30,31]. In addition, the size of the palladium particles corresponding to different reaction times is different. At 5 min of reaction, the particle size of the palladium particles in MXene@PdNPs5 was concentrated at 60 ± 5 nm as shown in Figure 3c. Further, as shown in Figure 3f, the particle diameter of the palladium particles in MXene@PdNPs20 is concentrated at 80 ± 5 nm. The experimental results show that the size of palladium particles increases with time, and the particle size of palladium particles of MXene@PdNPs60 composite is about 100 nm (Figure S3).

**Figure 4:**
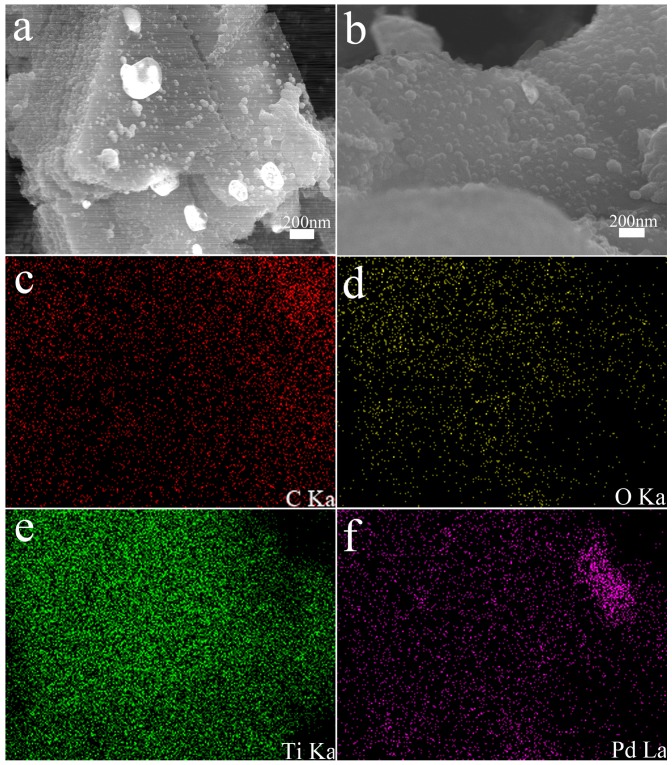
Representative SEM image of layered MXene@PdNPs5 (**a**) and MXene@PdNPs20 (**b**) with C/O/Ti/Pd elemental mapping (**c**–**f**).

As shown in Figure 4, the morphology of the MXene@PdNPs complex was characterized by SEM. Figure 4a,b depict the SEM structures of MXene@PdNPs5 and MXene@PdNPs20. The different nanostructures with increased reaction time can be seen from the images, and the PdNPs particles anchored on the surface of MXene are uniformly dispersed. The particle size gradually increased, while the number of particles increased. However, when the reaction time was long (such as 60 min), it was easy to cause aggregation of Pd particles (Figure S4). Through the elemental mapping analysis in the SEM, as shown in Figure 3c–f, the mapped images of MXene@PdNPs clearly show the presence of carbon, oxygen, titanium and palladium elements, further confirming the surface and interlayer loading. But most palladium particles are mainly distributed on the MXene surface.

**Figure 5:**
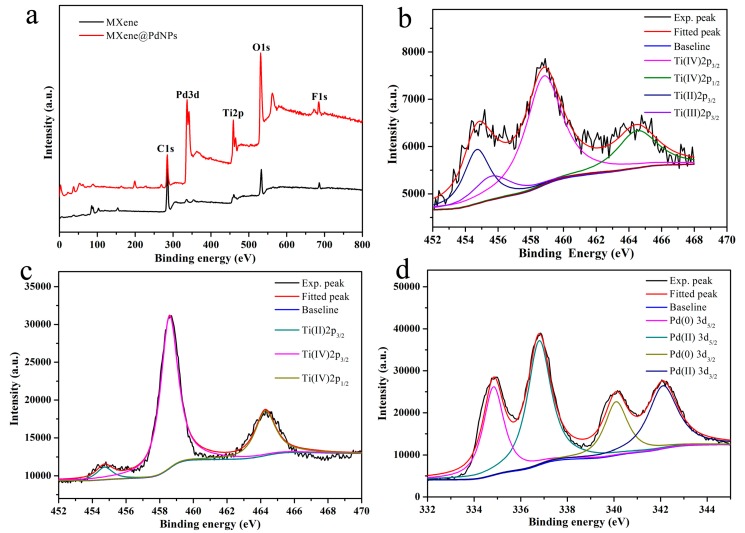
(**a**) XPS profiles of the MXene and MXene@PdNPs samples; (**b**) Ti2p in MXene; (**c**) Ti2p in MXene@PdNPs20; (**d**) Pd3d in the MXene@PdNPs20.

In addition, XPS is an effective tool to study the chemical state and element composition of various functional composites [32]. In order to study the mechanism of self-reduction, MXene and MXene@PdNPs20 composites were analyzed by XPS (Figure 5a). Ti (IV) 2p3/2 was assigned the obvious peak value of the original MXene sample at ~458.8 eV (Figure 5b). At the same time, the wide peak of 453.5–456.5 eV in Figure 5b indicates the existence of titanium with different valence states, including bivalent titanium at 454.8 eV and trivalent titanium at 455.8 eV [33,34,35,36,37,38,39]. In addition, in the self-reduction process of the composite material MXene@PdNPs20 (Figure 5c), the divalent titanium and trivalent titanium can be partially converted into tetravalent titanium (from 68% to 96% calculated from cumulative area of XPS peaks scanned from sample surface). As shown in Figure 5d, the binding energy of 334.8 eV and 340.1 eV corresponds to the Pd3d_5/2_ and the Pd3d_3/2_ component of the metal Pd(0) state with 44% percent calculated from cumulative area of XPS peaks, which confirms the presence of metal Pd [40]. In addition, the combined energy of 336.8 eV and 342.1 eV represent the divalent Pd(II) state with 56% percent calculated cumulative area of XPS peaks, suggesting the slight adsorption of PdCl2 salt on surface of MXene@PdNPs20 composite.

### 3.2. Dye Catalytic Performance of Nanocomposites

**Figure 6:**
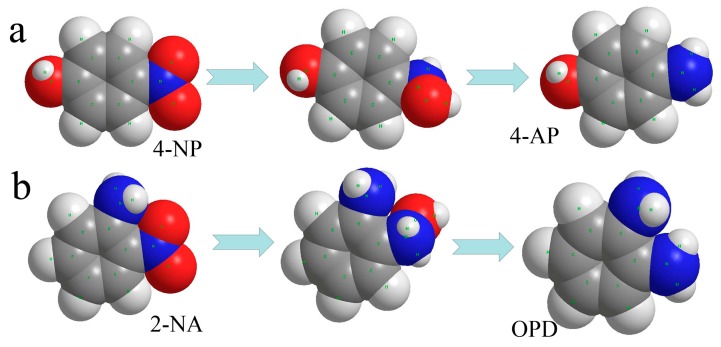
Spatial filling models of 4-NP transforming to 4-AP (**a**) and 2-NA transforming to OPD (**b**).

**Figure 7:**
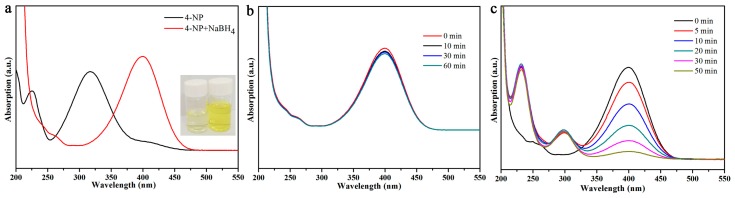
UV-vis absorption curves of 4-NP before and after adding NaBH_4_ aqueous solution (**a**); catalytic reduction of 4-NP with MXene (**b**) and PdNPs (**c**).

**Figure 8:**
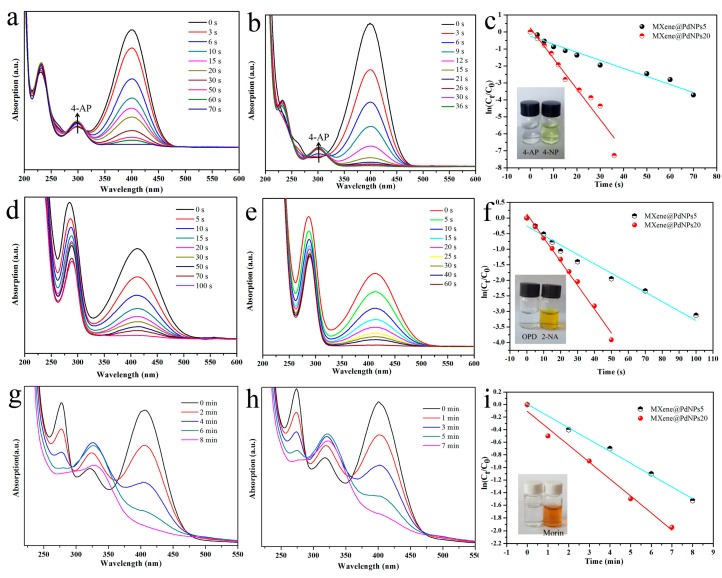
Catalytic reduction of 4-NP, 2-NA and morin by MXene@PdNPs5 composite material (**a**,**d**,**g**) and MXene@PdNPs20 composite material (**b**,**e**,**h**), respectively; (**c**,**f**,**i**) represent the corresponding relationship between the catalytic time and ln(C_t_/C_0_) under different catalysts.

Next, the obtained MXene@PdNPs composite was used as a new catalyst to study the catalytic properties of nitro compounds (4-NP, 2-NA) and morin (Figure 6) [41,42]. The catalytic activity of the synthesized MXene@PdNPs was evaluated by catalytic reduction of p-nitrophenol as a template reaction with an excess of NaBH_4_ solution. As shown in Figure 7, when NaBH_4_ was not added, the absorption peak of pure 4-NP was 317 nm. Under normal conditions, the 4-NP solution is pale yellow in neutral and acidic environments. After the addition of the NaBH_4_ solution, the solution immediately changed from light yellow to bright yellow due to the formation of p-nitrophenol ions. The absorption peak was 400 nm. As the reaction proceeded, an absorption peak of 4-AP appeared near 295 nm, at which time the solution was almost colorless. As shown in Figure 8a,b, MXene@PdNPs nanocomposites with different reaction times were added. The rate of catalytic reduction at 402 nm is different due to the different catalytic reaction rates owing to the addition of complexes. It is worth noting that due to the large initial concentration of NaBH_4_, the concentration of NaBH_4_ changes negligibly throughout the reaction. Therefore, we can think that the reaction rate of the whole reaction is independent of the concentration of NaBH_4_, and the reaction process can be regarded as the whole process of catalyzing 4-NP. Therefore, the pseudo-kinetic first equation can be used to evaluate the catalytic reaction rate. Since the absorbance of 4-NP is proportional to the concentration of the solution, we can think of the linear relationship between ln (C_t_/C_0_) (Ct represents the concentration at time t, C_0_ represents the initial concentration) and reaction time t. Using MXene@PdNPs as a catalyst to catalytically reduce 4-NP, the relationship between ln(C_t_/C_0_) and reaction time is shown in Figure 8c, and its linear relationship is consistent with the first kinetic equation. After calculation, the kinetic constant k of MXene@PdNPs5 as a catalyst for catalytic reduction of p-nitrophenol was 0.097 s^−1^. In addition, as comparison, the kinetic constant of p-nitrophenol catalyzed by MXene@PdNPs20 was 0.180 s^−1^. It is clear that the rate of MXene@PdNPs20 is more than twice of MXene@PdNPs5. More importantly, in comparison with other PdNPs systems [43,44,45,46] listed in Table 1, the present MXene@PdNPs nanocomposites exhibit excellent catalytic performance. It is worth mentioning that as the reaction time is extended, the catalytic performance is lowered. For example, the kinetic constant of the MXene@PdNPs60 nanocomposite used to reduce p-nitrophenol is 0.099 s^−1^ (Figure S5). Therefore, the time required to prepare the MXene@PdNPs5 complex on MXene surface is very short with relatively small size and number of the self-reducing palladium nanoparticles. Over time, when the reaction time reached 20 min, the MXene surface was loaded with more palladium nanoparticles and the dispersion was uniform, and the size of the particles gradually increased. When the reaction time reached 60 min, a large amount of agglomeration occurred in the PdNPs particles. Therefore, although small-sized particles contribute to catalysis, the particles are too small, so MXene@PdNPs20 exhibits higher catalytic activity. 

The catalytic performance of MXene@PdNPs catalyst for 2-NA reduction was studied by the same method. As shown in Figure 8d,e, it is worth mentioning that the absorption peak of the mixed solution did not change significantly after the addition of fresh NaBH_4_ to the 2-NA solution (Figure S6). After adding MXene@PdNPs5 catalyst for 100 s, 2-NA was completely reduced to o-phenylenediamine (OPD), and after adding MXene@PdNPs20 catalyst for 60 s, 2-NA was completely reduced to o-phenylenediamine (OPD). The experiments show that the addition of different catalysts has different catalytic capabilities for 2-NA. Obviously, it shows strong catalytic ability after adding the MXene@PdNPs20 catalyst. At the same time, morin is also catalyzed. In the whole process of catalyzing morin, the morin solution and the catalyst MXene @PdNPs were added to the cuvette in the buffer solution. It was then analyzed by UV-visible spectroscopy as shown in Figure 8g,h. With the increase of time, the peak of mulberry pigment centered on 410 nm gradually became smaller and eventually became absent, indicating that the morin pigment gradually degraded. It exhibits strong catalytic performance, and the ability to catalyze morin is considerably weaker than that of catalyzing 4-NP and 2-NA. In summary, MXene@PdNPs composites exhibit higher catalytic efficiency for 4-NP, especially MXene@PdNPs20 nanomaterials.

**Figure 9:**
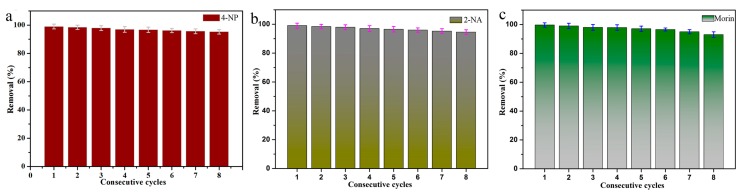
The cyclic catalytic capacity of MXene@PdNPs20 composite for the reduction of 4-NP (**a**), 2-NA (**b**) and morin (**c**).

The stability and recyclability of the catalyst is another aspect of evaluating the catalyst. Therefore, it is important and necessary to explore the repeated catalytic ability of MXene@PdNPs catalyst for 4-NP, as shown in Figure 9a. For catalytic 4-NP, the catalysis efficiency of MXene@PdNPs20 catalyst after eight cycles of repeated catalytic reduction (reach 1 min as compared time point) was still as high as 94%. The used composite material was centrifuged, and the supernatant was decanted. The precipitate was washed several times with ethanol and ultrapure water and dried again in vacuum. XRD test results (Figure S7) of the used MXene@PdNPs20 composite after eight catalytic cycles remained unchanged and confirmed the stability of the MXene-PdNPs composite. Referring to previous literature, the few decrements in catalytic efficiency were reasonably attributed to the attachment of organics on the surface of catalyst and the loss of palladium particles on the catalyst surface during the washing of MXene@PdNPs with ethanol and ultrapure water [15]. In summary, the results indicate that the MXene@PdNPs catalyst has high catalytic activity, stability and recyclability. The new composite materials of MXene@PdNPs provide broad application prospects for new catalyst research fields such as dye catalytic degradation and sewage treatment [47,48,49,50,51,52,53,54,55], and expand ideas for the research of similar self-assembled nanocomposites [56,57,58,59,60,61,62,63,64,65].

## 4. Conclusions

In summary, a new MXene@PdNPs nanocomposite was prepared by a self-reduction strategy in this experiment. The catalytic properties of the prepared novel nanocomposite MXene@PdNPs vary depending on the time of the self-reduction reaction. MXene@PdNPs metal Pd particle nanocomposites with different reaction times were prepared by different self-reduction reaction times. MXene@PdNPs materials exhibit excellent reducing power by catalytic reactions of compounds such as 2-NA, 4-NP and morin. In particular, MXene@PdNPs20 exhibited high catalytic effects on 4-NP and 2-NA, and the first-order rate constants of the catalysts were 0.180 s^−1^ and 0.089 s^−1^, respectively. The experimental results showed that the prepared nanocomposite MXene@PdNPs20 has the best catalytic effect. In addition, after eight consecutive catalytic cycles, the conversion of the catalytic 4-NP was still greater than 94%, with high catalytic activity, stability and recyclability. Therefore, this work provides a new method for the preparation of MXene-based nanocatalysts and composites, while the research of self-assembled MXene@PdNPs nanocomposites has important potential value for environmental management and sustainable development of composite materials.

## Figures and Tables

**Table 1:** Comparative characteristics and catalytic performance of PdNPs-based materials.

No.	Materials	Kinetic Constant k/s^−1^	Characteristics	Refs
1	Pd@B.tea NPs	0.00059	Natural bioactive black tea extract reducing agent	[43]
2	CN-supported PdNPs nanohybrids	0.00570	Cellulose nanocrystals as support matrix and reducing agent	[44]
3	AuPdNPs/graphene nanosheets (GNs)	0.01445	Bimetallic nanoparticles, monodisperse method, graphene nanosheet carrier	[45]
4	Pd/magnetic porous carbon (MPC)	0.01200	Porous carbon composite catalyst carrier, magnetic separation	[46]
5	MXene@PdNPs	0.1800	self-reduction, simple method	Present work

## Supplementary Materials

The following are available online at https://www.mdpi.com/2079-4991/9/7/1009/s1, Figure S1. XRD patterns (a) and TG curves (b) of prepared MXene composites. Figure S2. HRTEM image of the PdNPs catalyst. Figure S3. TEM image of the prepared MXene@PdNPs60 (a) and high-resolution TEM image of Pd nanoparticles (b). Figure S4. SEM image of layered MXene@PdNPs60. Figure S5. Catalytic reduction of 4-NP by MXene@PdNPs60 composite material (a) and (b) represent the corresponding relationship between the catalytic time and ln(Ct/C0). Figure S6. UV-vis absorption curves of 2-NA before and after adding NaBH4 aqueous solution. Figure S7. XRD patterns of prepared MXene@PdNPs20 before and after reduction of 4-NP with eight cycles.
