# Supplementary material for: Highly Efficient Catalytic Performances of Nitro Compounds and Morin via Self-Assembled MXene-Pd Nanocomposites Synthesized through Self-Reduction Strategy
Source: Nanomaterials (Basel). 2019 Jul 12;9(7):1009. doi: 10.3390/nano9071009 (PMC6669661; doi:10.3390/nano9071009)
Supplement: Supplementary file 1 [file nanomaterials-09-01009-s001.pdf]

Supporting information

Highly Efficient Catalytic Performances of Nitro Compounds and Morin via Self-Assembled MXene-Pd Nanocomposites Synthesized through Self-Reduction Strategy

Juanjuan Yin [1,2], Lun Zhang [2], Tifeng Jiao [1,2,*], Guodong Zou [1], Zhenhua Bai [3], Yan Chen [2], Qingrui Zhang [2,*], Meirong Xia [2], Qiuming Peng [1]

[1] State Key Laboratory of Metastable Materials Science and Technology, Yanshan University, Qinhuangdao 066004, China; jjy1729@163.com (J.Y.); zouguodong2015@stumail.ysu.edu.cn (G.Z.); pengqiuming@ysu.edu.cn (Q.P.)

[2] Hebei Key Laboratory of Applied Chemistry, School of Environmental and Chemical Engineering, Yanshan University, Qinhuangdao 066004, China; lawleit123@163.com (L.Z.); chenyan@ysu.edu.cn (Y.C.); xmr0125@126.com (M.X.);

[3] National Engineering Research Center for Equipment and Technology of Cold Strip Rolling, Yanshan University, 438 West Hebei Street, Qinhuangdao 066004, P. R. China; bai_zhenhua@aliyun.com (Z.B.)

* Correspondence: tfjiao@ysu.edu.cn (T.J.); zhangqr@ysu.edu.cn (Q.Z.); Tel.: +86-335-805-6854 (T.J.)

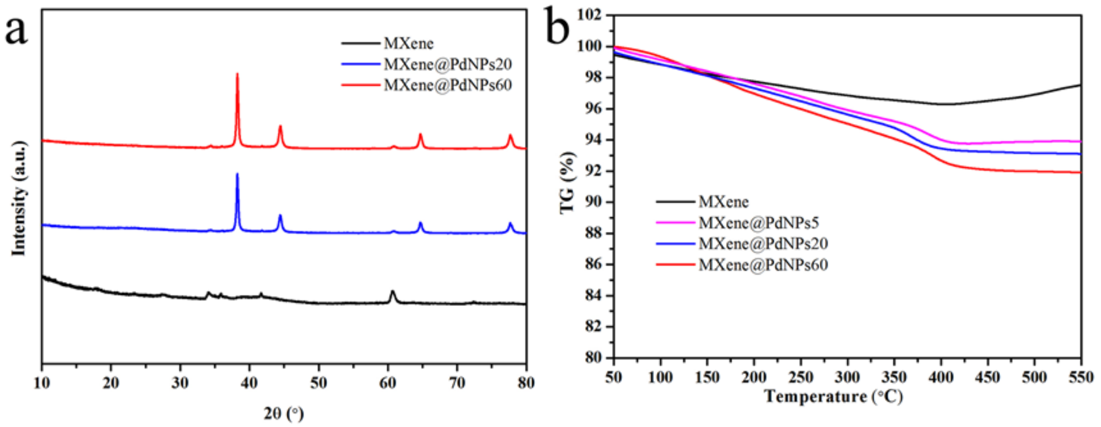

Figure S1. XRD patterns (a) and TG curves (b) of prepared MXene composites.

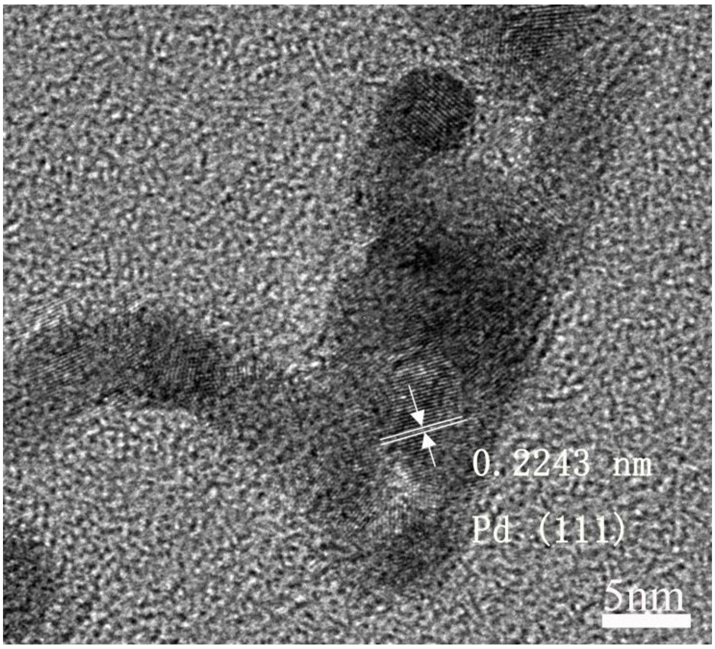

Figure S2. HRTEM image of the PdNPs catalyst.

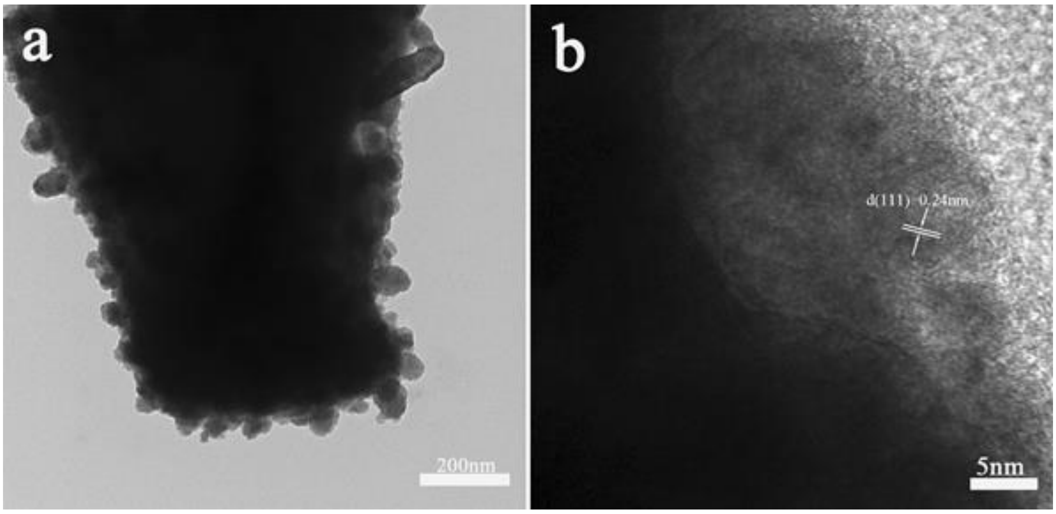

Figure S3. TEM image of the prepared MXene@PdNPs60 (a) and high-resolution TEM image of Pd nanoparticles (b).

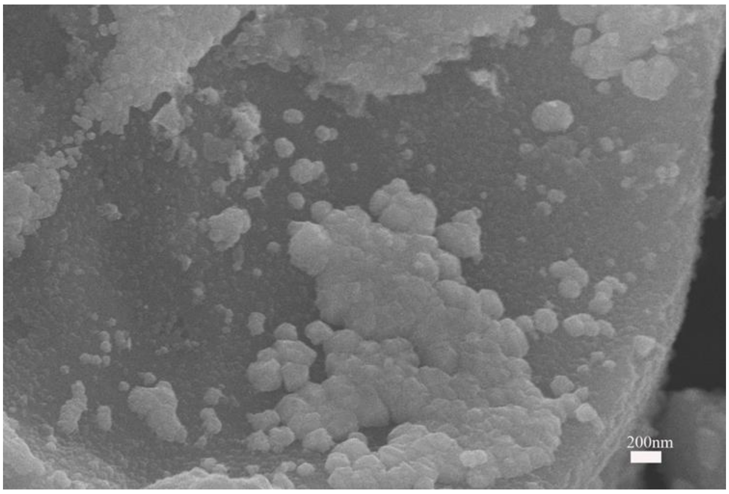

Figure S4. SEM image of layered MXene@PdNPs60.

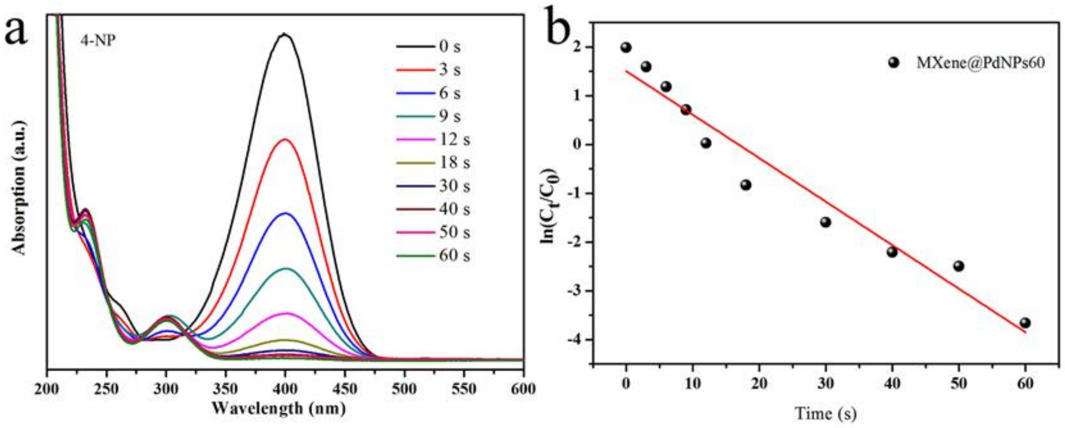

Figure S5. Catalytic reduction of 4-NP by MXene@PdNPs60 composite material (a) and (b) represent the corresponding relationship between the catalytic time and ln(Ct/C0).

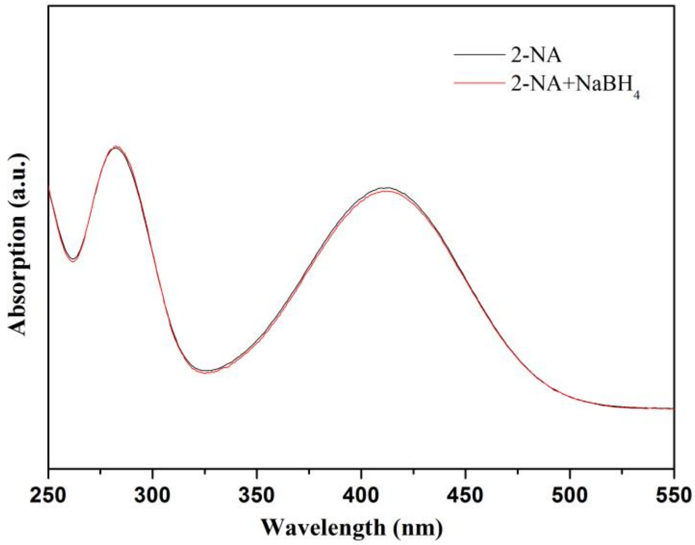

Figure S6. UV-vis absorption curves of 2-NA before and after adding $NaBH_4$ aqueous solution.

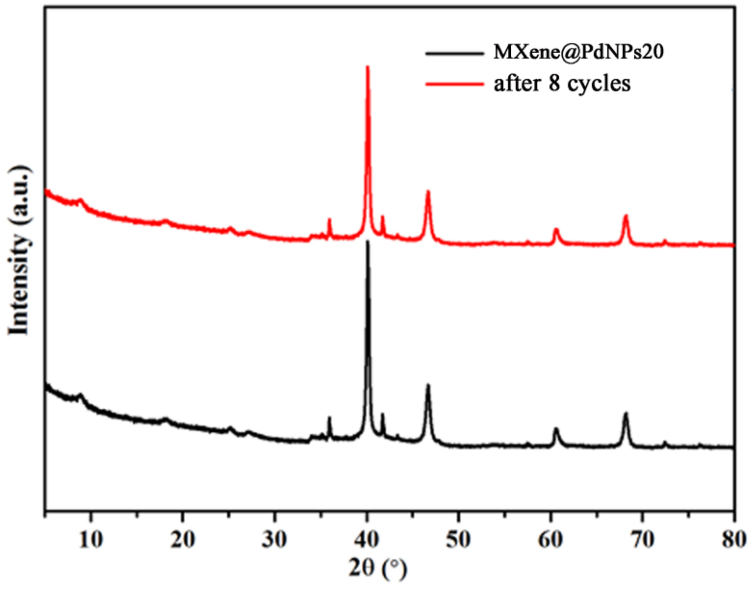

Figure S7. XRD patterns of prepared MXene@PdNPs20 before and after reduction of 4-NP with 8 cycles.
